# Adaptation of Social Health Care Tools From Paper-Based to Web-Based Formats: Scoping Review

**DOI:** 10.2196/88631

**Published:** 2026-08-06

**Authors:** Charlèss Dupont, Eva Savolainen, Petter Fjällström, Malin Eneslätt

**Affiliations:** 1 End-of-Life Care Research Group, Vrije Universiteit Brussel, Brussels, Belgium; 2 Department of Health, Education and Technology, Luleå University of Technology, Universitetsområdet, Luleå, Norrbotten, Sweden, 46 722139392; 3 Department of Community Medicine and Rehabilitation, Umeå University, Umeå, Västerbotten, Sweden; 4 Department of Learning, Informatics, Management and Ethics, Karolinska Institutet, Stockholm, Stockholm, Sweden

**Keywords:** digital health, user-centered design, implementation, scoping review, eHealth, usability

## Abstract

**Background:**

The transition of social health care tools from paper-based to web-based formats is increasingly common in social and health care settings. While digital delivery may improve accessibility, flexibility, and scalability, such transitions require careful adaptation of content, design, and workflows. However, evidence on how these transitions are carried out in practice, and which challenges and strategies are involved, remains fragmented.

**Objective:**

This scoping review aimed to map how social health care tools have transitioned from paper-based to web-based formats and to synthesize evidence on tool characteristics and target groups, adaptations made during the transition, strategies and methodologies used, and reported challenges and solutions.

**Methods:**

A scoping review was conducted following the Arksey and O’Malley framework. Systematic searches were performed in PubMed, PsycINFO, Scopus, and Web of Science to identify English-language empirical studies describing the transition of social health care tools from paper-based to web-based formats. Social health care tools were defined as technologies supporting communication, reflection, or informed decision-making among patients, family caregivers, and/or health care professionals. Two reviewers independently screened studies and extracted data using a standardized form. Targeted gray literature searches were conducted to supplement missing information about the included tools. Findings were synthesized narratively and presented in tables.

**Results:**

Eleven tools were included. Most were delivered as web-based platforms (n=8), with additional tools delivered as mobile apps (n=2) or in a game-based format (n=1). The tools targeted diverse populations, including people with chronic or life-limiting conditions, family caregivers, adolescents, parents, pregnant women, and health care professionals. Reported reasons for transitioning to digital formats included improving accessibility and reach (n=7), enhancing user engagement through interactive features (n=5), and increasing efficiency or scalability (n=3). Across tools, core content was largely retained, while adaptations mainly focused on format and delivery, such as multimedia elements, interactive modules, tailored feedback, and usability improvements. User-centered and participatory design approaches were commonly reported, sometimes combined with iterative or agile development methods. Common challenges included usability issues, low engagement, technical integration difficulties, administrative delays, and resource constraints, with solutions focusing on iterative redesign, clearer instructions, increased interactivity, and hybrid paper-digital approaches.

**Conclusions:**

This scoping review examines the process of transitioning social health care tools from paper-based to web-based formats, rather than focusing solely on effectiveness outcomes. It synthesizes how adaptations are made, which strategies are used, and which challenges arise during digitalization. The findings identify common adaptation patterns and gaps in reporting, particularly regarding target group–specific design and equity considerations. These insights may support developers, researchers, and policymakers in planning more usable, inclusive, and context-sensitive digital transitions in social and health care settings.

## Introduction

In recent years, a notable paradigm shift has occurred with the transition of tools in social and health care settings from traditional paper-based to interactive online formats [1,2]. For instance, digital tools now replace paper diaries, allowing patients to enter their medical information from home in real time [3]. This enhances data accuracy and timeliness, enabling health care providers to offer more personalized and proactive care [1,4].

This transition takes place within a broader process of digital transformation in health and social care systems. Recent literature emphasizes that digitalization is not only about introducing new technologies but also about adapting existing tools and care processes to digital environments [5,6]. However, while much research has focused on the development and evaluation of new digital interventions, less attention has been paid to how existing paper-based tools are systematically transitioned to web-based formats. It is crucial to recognize that this shift necessitates careful adaptation to ensure that online tools are perceived and used effectively [7,8]. Unlike paper-based tools, online tools may present information differently and require users to navigate through various digital interfaces [7].

Despite the advantages, such as improved accessibility and real-time updates, transitioning from paper to digital formats may present several challenges. These include user resistance, digital literacy issues, accessibility barriers, and the need for technical support and training [9,10]. Recent studies have further highlighted ongoing digital inequalities in health care access, particularly among older adults and socially vulnerable groups [11-13].

Individual studies have reported mixed results regarding the effectiveness of transitioning from paper-based to online formats [14,15]. In some cases, digital tools are designed to closely resemble their paper versions to support familiarity. However, this approach may not always be suitable in an online environment or for different target groups. Importantly, the transition from paper to web-based formats is not merely a technical conversion; it often involves conceptual and structural changes that may affect usability, user engagement, and accessibility [16]. Moreover, these studies typically evaluate outcomes after implementation and provide limited insight into how and why specific adaptations were made during the transition process.

There is still limited knowledge about how existing tools are adapted for online use, which specific modifications are made during this process, and how these adaptations influence their usability and accessibility. To our knowledge, no comprehensive overview has systematically mapped which tools in social health care settings have undergone such transitions, how these transitions were performed, and what challenges and solutions were reported. A clearer understanding of these processes is needed to better inform future transitions and to avoid repeating common design or implementation problems.

Therefore, this paper explores how tools used in social and health care settings (hereafter called social health care tools) have been transitioned from paper-based to web-based formats. It examines their characteristics, strategies, methodologies, challenges, and solutions:

1. Which social health care tools have shifted from paper-based to web-based formats, and what are their target groups, countries of development, formats, aims, and the reasons behind the transition?
2. What specific changes were made when transitioning social health care tools from traditional paper-based formats to web-based platforms?
3. What strategies and methodologies were used to transition the tools from paper-based formats to online platforms?
4. What were the key challenges encountered during the transition, and how were these challenges addressed?

## Methods

### Design and Frameworks

Scoping review studies map key concepts and evidence in a research area and can stand alone, particularly in complex or underreviewed domains [17]. For our scoping review focusing on studies or reports on transitioning social health care tools from traditional paper-based to web-based formats, we adopted the methodological framework outlined by Arksey and O’Malley [17], with no critical appraisal of the studies. This framework involves 5 stages: identifying the research question, locating relevant studies, selecting studies, charting the data, and collating and summarizing the results. The PRISMA-ScR (Preferred Reporting Items for Systematic Reviews and Meta-Analyses Extension for Scoping Reviews) checklist guided the reporting of this scoping review (Checklist 1) [18]. In addition, the search strategy and reporting were documented according to the PRISMA-S (Preferred Reporting Items for Systematic Reviews and Meta-Analyses Literature Search Extension) extension to strengthen transparency and reproducibility of each component of the search [18]. No prior protocol was established or published.

### Eligibility Criteria

Eligibility criteria were defined using the Population-Concept-Context framework recommended for scoping reviews [19]. Studies were eligible if they reported on the following criteria:

1. Social health care tools used by patients, family caregivers, health care professionals, or related stakeholders (Population).
2. The transition of a tool from a paper-based to a web-based or internet-based format (Concept).

Social health care tools were defined as structured instruments intended to support communication, reflection, informed decision-making, self-management, coordination, or psychosocial support within social or health care settings.

Empirical qualitative, quantitative, or mixed methods studies conducted in any clinical, community, or organizational context were eligible (Context), provided they reported on adaptations made during the transition process and/or strategies, methodologies, challenges, or solutions related to digitalization.

Studies were excluded if they did not involve information on the paper-to-web transition, focused exclusively on physical health without a social or communicative component, were not original empirical research, or described transitions in tools delivered via non–internet-based formats (eg, CD-ROM only). Only English-language publications were included. Gray literature was consulted solely to supplement missing information on included tools.

### Information Sources and Search Strategies

To ensure a thorough exploration of the relevant literature, we used a comprehensive search strategy. This strategy involved systematic searches of electronic databases (Multimedia Appendix 1), including PubMed, PsycINFO, Scopus, and Web of Science, to identify peer-reviewed articles and scholarly publications. The full electronic search strategies for each database, including all search terms, Boolean operators, applied limits, and the date on which each search was performed, are provided in Multimedia Appendix 1 in accordance with PRISMA-S [18]. Searches were limited to English-language publications.

In addition, we supplemented the database searches with backward citation searching, consistent with Joanna Briggs Institute recommendations for scoping reviews [20]. After full-text screening, the reference lists of all included articles were examined to identify additional potentially relevant sources describing the transition of social health care tools from paper-based to web-based formats. Records identified through this process were retrieved and assessed against the same eligibility criteria as database-derived records. Eligible studies identified through citation searching were included in the review and integrated into data extraction and synthesis.

Additionally, recognizing the potential significance of nonacademic sources, we searched for extra information on the included tools in gray literature, such as conference proceedings, reports, theses, and dissertations. Two researchers (CD and ES) independently conducted the searches, using the name of the included tool or other identifiers (author names, institution names, etc). Each researcher conducted the searches on separate computers, ensuring privacy by clearing browser history and cookies and avoiding logins to Google accounts. The content of the search results, such as executive summaries, was examined to determine missing information on adapting a web-based support tool, such as the adaptation process and challenges encountered during the transition.

### Selection of Sources

First, CD searched all aforementioned databases and screened records based on their titles. All records retrieved from the database searches were imported into an Excel file, where duplicates were removed, and the deduplicated records were imported to Rayyan software. After deduplication, 2 reviewers (CD and ES) independently screened the titles and abstracts of retrieved articles for relevance to the research questions and eligibility criteria. Full texts of potentially relevant articles were assessed for final inclusion. Any discrepancies in screening and selection were resolved through discussion and consensus with all authors. Records identified through backward citation searching were screened using the same process.

For each included tool, targeted gray literature searches (eg, via Google) were performed using the tool name, author names, or institutional affiliations to retrieve additional publicly available information on the adaptation process. These searches were used solely to supplement missing details when needed and did not lead to the inclusion of additional tools.

### Data Charting, Items, and Synthesis

Data extraction was conducted independently by 2 researchers (CD and ES) using a standardized data extraction form. The form was piloted and refined before full extraction. Data were extracted to address the 4 research questions, including study and tool characteristics, target groups, reasons for transition, adaptations made during the transition, development methodologies and strategies, and reported challenges and solutions. Where relevant, targeted gray literature searches were used to supplement missing information on included tools. Extracted data were synthesized per research question and presented narratively and in tables.

Although eligibility screening was performed at the level of published papers, synthesis was conducted at the level of the tool. This approach was chosen because multiple publications may describe the same tool, and the aim of this review was to analyze tool characteristics and adaptation processes rather than publication outcomes.

## Results

### Selection Process

**Figure 1.**
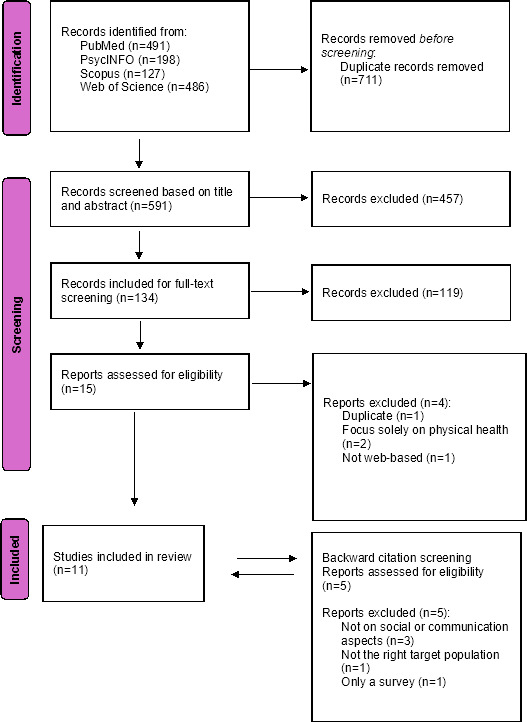
PRISMA (Preferred Reporting Items for Systematic Reviews and Meta-Analyses) flowchart of the selection process.

The search across academic databases resulted in a total of 1302 peer-reviewed articles: 491 from PubMed, 486 from Web of Science, 198 from PsycINFO, and 127 from Scopus. All results were imported into an Excel file to remove duplicates. After removing duplicates, CD screened the records based on titles, and 591 articles remained. These articles were screened by CD and ES based on abstracts, after which 134 articles were selected for full-text review. Based on this full-text screening, 15 tools were initially included. However, following a discussion among all authors on the eligibility criteria and the included tools, 4 of these tools were excluded. One was a duplicate already included from another source, 2 focused exclusively on physical health and did not address social or communicative aspects of health care, and 1 tool was delivered via CD-ROM rather than a web-based format. No additional tools were included based on backward citation searching. An overview of the selection process is shown in Figure 1.

### Characteristics of Social Health Care Tools Transitioned From Paper-Based to Web-Based Formats

Table 1 provides an overview of the 11 included social health care tools (hereafter called tools), including their aims, formats, target groups, countries of development, and reported reasons for the transition from paper-based to web-based delivery [21-31]. Most tools were developed as web-based platforms (n=8; [22,23,25-27,30,31]), with newer tools delivered as mobile apps (n=2; [24,29]) or in a game-based format (n=1; [21]).

**Table 1.** Characteristics of social health care tools transitioned from paper-based to web-based formats.

Study	Tool name	Target population	Aim of the tool	Country of development	Digital format	Reason for transition
Bayley and Brown [21], 2015	What Should We Tell the Children? (WSWTTC)	Parents of children and adolescents	Support parent-child communication about sex and relationships	United Kingdom	Game-based	Reduce participation barriers and increase engagement
Honary et al [22], 2018	Relatives Education And Coping Toolkit (REACT)	Relatives of people with psychosis or bipolar disorder	Provide informational and emotional support	United Kingdom	Web-based	Improve accessibility and scalability
Kingston et al [23], 2015	Not specified	Pregnant women	Support mental health screening	Canada	Web-based	Improve efficiency and reduce disclosure barriers
Lee et al [24], 2023	Promoting Independence in Dementia (PRIDE) app	People with mild dementia	Support self-management and independence	United Kingdom	App	Improve usability and accessibility
Monnet et al [25], 2023	Levenswensen kaarten	People with dementia and family caregivers	Support advance care planning	Belgium	Web-based	Improve accessibility and usability
Peels et al [26], 2012	Active Plus	Adults aged ≥50 years	Promote physical activity	Netherlands	Web-based	Improve reach and enable tailoring at scale
Staffileno et al [27], 2015	Not specified	Young African American women	Promote lifestyle behavior change	United States	Web-based	Improve accessibility and engagement
Stille et al [28], 2015	Parent-Provider Partnerships in Referral Communication (P3RC)	Health care professionals and parents	Improve referral communication	United States (Colorado, Oklahoma)	Web-based	Improve workflow integration and efficiency
Svarre et al [29], 2017	Housing Enabler app	Health care professionals	Assess environmental accessibility	Denmark	App	Improve data accuracy and efficiency
Van Goethem et al [30], 2023	Web-based program for Family involvement, Optimistic outlook, Coping effectiveness, Uncertainty reduction, and Symptom management (iFOCUS)	People with advanced cancer and family caregivers	Support emotional well-being and self-efficacy	Belgium, Netherlands, Denmark, Italy, United Kingdom, and Ireland	Web-based	Improve accessibility and self-management
Whitehouse et al [31], 2013	TickiT	Adolescents in health care settings	Support psychosocial screening	Canada	Web-based/App	Improve efficiency and youth engagement

The tools addressed a range of aims within social and health care settings, with most focusing on psychosocial support, self-management, communication, or screening. Target groups included patients with chronic or life-limiting conditions, family caregivers, adolescents, parents, pregnant women, and health care professionals. While several tools were primarily intended for patients or caregivers, a smaller subset targeted health care professionals and focused on clinical or administrative processes.

Tools were developed across multiple countries, predominantly in Europe and North America. Across studies, the most frequently reported reasons for transitioning to digital formats were to improve accessibility and availability (eg, enabling use from home and outside clinical appointments), enhance user engagement through interactive features (eg, videos, quizzes, tailored feedback, automated scoring, or reminders), and increase efficiency or scalability. In some cases, external contextual factors, such as the COVID-19 pandemic, were reported as contributing to the decision to digitalize existing tools.

In general, information on the transition process was limited. Information provided described the transition from paper-based to web-based formats as an approach to address limitations of paper tools, such as limited opportunities for remote use, reduced flexibility, or challenges in routine implementation, while preserving the core purpose and content of the original tools.

### Changes Made During the Transition

A range of adaptations were made during the transition from paper-based to web-based formats, tailored to the function, delivery setting, and target audience of each intervention (see Table 2). In 5 tools [21,22,24,26,27], the original intervention content was largely retained, while the format and delivery were modified to include digital features such as videos, online modules, quizzes, or interactive exercises. In 3 tools [21,22,30], the structure and delivery of sessions were also redesigned, for example, moving from face-to-face or nurse-led delivery to self-guided or asynchronous modules [30]. Five tools [21,22,25,27,31] integrated features that allowed users to navigate content adaptively or receive tailored feedback, such as adaptive navigation, customized feedback, or emotionally responsive content. Design changes to improve usability, such as font adjustments, simplified navigation, and tailored login systems, were also reported.

**Table 2.** Overview of adaptations made during the transition from paper-based to web-based formats.

Study	Original paper-based feature	Web-based adaptation and purpose of change
Bayley and Brown [21], 2015	“What Should We Tell the Children?” group program with exercises to support parent-child communication about sex and relationships.	Converted into an online serious game with first-person role-play, interactive quizzes, and feedback. Purpose: to test the acceptability of converting a group program into an online serious game.
Honary et al [22], 2018	Relatives Education And Coping Toolkit (REACT) booklet: modular print toolkit for relatives of people with psychosis or bipolar disorder.	Converted to an online intervention with modules, resources, forums, and brief video clips. Purpose: to provide structured information and support in a web-based format.
Kingston et al [23], 2015	Paper-based maternal mental health screening tools.	Developed secure tablet and web-based e-screening for mental health, comparing perceptions of web-based vs paper-based formats. Purpose: to evaluate the acceptability and impact of web-based screening.
Lee et al [24], 2023	Promoting Independence in Dementia (PRIDE) manual-based psychosocial self-management program for mild dementia, including facilitator sessions.	Developed a web-based self-management app with similar session structure and usability enhancements (layout, login, and icons). Purpose: to support digital access and tailor the interface for users with dementia.
Monnet et al [25], 2023	Levenswensen (Life Wishes) cards supporting discussion on advance care planning in person.	Integrated into a web-based advance care planning tool designed for people with dementia and their family caregivers; developed through a user-centered iterative design process. Purpose: to create an online platform for advance care planning discussions.
Peels et al [26], 2012	Active Plus computer-tailored printed intervention to promote physical activity among adults aged ≥50 years.	The intervention was translated to a web-based format using the RE-AIM (Reach, Effectiveness, Adoption, Implementation, and Maintenance) framework; content remained the same, but interactive outputs differed (eg, static pictures → videos, printed maps → embedded maps, added hyperlinks). Purpose: to provide tailored advice via the internet with similar core content.
Staffileno et al [27], 2015	In-person lifestyle intervention using manuals, tracking logs, and group sessions for physical activity and dietary change among young African American women.	Restructured into digital modules with multimedia components (videos, digital logs, and discussions) accessible on multiple devices. Purpose: to make behavior change content available electronically and support user self-direction.
Stille et al [28], 2015	Parent-Provider Partnerships in Referral Communication (P3RC) structured form to improve provider communication about referrals via paper or traditional processes.	Adapted to an electronic environment, integrated with digital clinical workflows and electronic health record systems. Purpose: to support electronic communication within practice systems.
Svarre et al [29], 2017	Housing Enabler paper instrument assessing environmental barriers.	Developed a digital app with item navigation, automated scoring, and data export. Purpose: to support data capture and usability in digital form.
Van Goethem et al [30], 2023	Original FOCUS program: a face-to-face psychoeducational dyadic intervention for people with advanced cancer and family caregivers.	Developed a self-managed web-based psychoeducational eHealth program (iFOCUS) using Scrum methodology for iterative development and usability testing. Purpose: to describe development and evaluate the acceptability of the web-based program.
Whitehouse et al [31], 2013	Paper-based Adolescent Screening Questionnaire for psychosocial health screening.	Converted to an eHealth platform (TickiT) cocreated with youth; adaptations included format changes for digital delivery. Purpose: to design and evaluate an online screening platform.

### Strategies and Methodologies Used in the Transition Process

All 11 tools described a structured process to support the transition from paper-based to web-based formats, although the level of detail and the guiding frameworks varied (see Table 3). User-centered design was explicitly reported in 4 studies [22,24,25,30], while several additional tools applied closely related participatory or cocreation approaches [25,29,31]. This included strategies that involved users, such as patients, caregivers, and health care professionals, in interviews, workshops, think-aloud sessions, and cocreation exercises to ensure the digital tools were aligned with users’ needs and practical contexts of use. These approaches involved patients, caregivers, health care professionals, or other stakeholders in workshops, interviews, think-aloud sessions, prototype reviews, and co-design activities to ensure alignment with users’ needs and real-world contexts of use.

**Table 3.** Methodologies and strategies used to support the transition from paper-based to web-based social health care tools.

Study	Methodology (guiding approach or framework)^a^	Strategies (concrete methods or activities)^a^	Description
Bayley and Brown [21], 2015	Intervention mapping; theory-informed adaptation	Formative research; scenario scripting; role-play game development; message-framing survey; pre-post feasibility evaluation	Core components of the original program were reviewed for theoretical alignment and digital feasibility. Formative research informed development of an interactive role-play format
Honary et al [22], 2018	User-centered design	Workshops; prototype review; heuristic evaluation; think-aloud testing; short field testing	The web-based intervention was developed through workshops and iterative prototype evaluation, including heuristic and usability testing to inform refinements
Kingston et al [23], 2015	Randomized controlled trial as evaluation design	Digital implementation of validated tools; random assignment; secure data management	The transition to digital screening was evaluated using a randomized design comparing paper-based and electronic formats within clinical practice
Lee et al [24], 2023	User-centered design; agile development	Stakeholder consultations; wireframe reviews; think-aloud testing; iterative sprints	The app was developed through stakeholder engagement and iterative refinement, incorporating usability testing across development stages
Monnet et al [25], 2023	User-centered and participatory design; iterative agile development; Medical Research Council (MRC) framework	Cocreation; think-aloud testing; surveys and interviews; design sprints	The advance care planning tool was developed through participatory design and iterative testing with stakeholders, integrating feedback across development phases
Peels et al [26], 2012	Mixed methods pretesting and usability evaluation	Questionnaires; observations; expert and nonexpert usability testing; iterative refinement	The intervention was refined through quantitative and qualitative pretesting, including usability assessments and feedback-driven revisions
Staffileno et al [27], 2015	Phased development and feasibility evaluation	Formative sessions; digitization into web modules; usability testing; beta testing	The intervention was digitized through staged development, incorporating formative feedback and iterative usability testing prior to implementation
Stille et al [28], 2015	Practice-based implementation adaptation to electronic environments	Pilot testing; collaboration with IT teams; workflow mapping; electronic integration	The intervention was adapted for electronic clinical environments through pilot testing and integration into existing digital workflows
Svarre et al [29], 2017	Participatory research design	Observations and interviews; co-design workshops; prototype development; usability testing; iterative refinement	The digital assessment tool was developed through participatory workshops and prototype testing, incorporating iterative usability feedback
Van Goethem et al [30], 2023	User-centered design; Scrum/agile methodology	Iterative development sprints; stakeholder consultations; usability testing; feedback loops	The web-based program was developed using an iterative agile process with ongoing stakeholder input and usability testing to guide refinements
Whitehouse et al [31], 2013	Cocreation; iterative design and evaluation; mixed methods feasibility study	Stakeholder cocreation; pilot feasibility testing	TickiT was developed through iterative cocreation with youth and health care stakeholders, followed by feasibility evaluation in clinical settings

a “Methodologies” refer to overarching development approaches or guiding frameworks (eg, user-centered design, participatory design, Agile/Scrum, Intervention Mapping), whereas “strategies” refer to concrete activities used within these approaches (eg, cocreation workshops, interviews, think-aloud usability testing, pilot testing, and heuristic evaluation).

Iterative development processes were common. Agile or Scrum-based methodologies were described in 3 studies [24,25,30], while other tools incorporated iterative prototype testing and refinement cycles supported by usability testing and stakeholder feedback [22,29,31]. “What Should We Tell the Children?” (WSWTTC) applied Intervention Mapping to ensure theoretical alignment and structured adaptation to a game format [21]. Other studies reported phased development, mixed methods pretesting, pilot implementation, or randomized evaluation designs to assess the feasibility, usability, or effectiveness of the digital adaptation [23,26,28]. While these studies did not specify a formal design framework, they described systematic evaluation strategies to support and assess the transition process.

### Challenges Encountered and Solutions Implemented

Usability was a frequently reported barrier (n=6; [22,25-27,30,31]), especially in tools aimed at older adults or people with limited digital skills. Users experienced difficulties with navigation, unclear instructions, small fonts, or confusing icons. Other tools (n=2; [21,32]) struggled with user engagement, reporting high dropout rates or limited adherence, often linked to overly long text, limited interactive elements (eg, quizzes or exercises), or cognitive burden. Technical challenges (n=4; [22,28,30,31]) included bugs, login issues, and difficulties integrating the tools into existing clinical systems. Administrative and implementation delays, such as navigating complex information and communication technology systems or securing institutional approvals, further complicated the transition process.

To address usability issues, several teams adopted iterative design strategies informed by user testing [22,26,30,31], resulting in clearer interfaces, instructional videos, customizable font sizes, and more intuitive navigation. Tools with engagement problems revised their content and structure, for example, reducing text volume, simplifying feedback messages, and adding interactive components such as videos, exercises, or tailored feedback. Technical bugs were tracked and resolved through internal reporting systems and regular meetings with development teams. In some tools [24,26,29], more pragmatic solutions, such as combining digital and print components or retaining the original structure with only minor digital adaptations, were adopted to stay within project constraints.

## Discussion

### Principal Findings

This scoping review examined how social health care tools have been adapted from paper-based to web-based formats. While the content of most tools remained largely unchanged, the transition often involved adding specific digital features, such as videos, online modules, quizzes, interactive exercises, adaptive navigation, and tailored feedback. User-centered and iterative development approaches were central to the transition processes, helping to identify usability barriers and refine the layout, navigation, and content presentation. At the same time, the shift to a web-based format introduced new challenges, particularly related to digital literacy, login procedures, navigation difficulties, and sustained user engagement. Most solutions to mitigate these challenges were pragmatic and context-specific, including simplifying interfaces, reducing text volume, adjusting font sizes, and combining digital components with existing workflows, highlighting the importance of adaptable and context-sensitive design when moving tools into digital environments.

This scoping review contributes to the field by focusing on the transition process from paper-based to web-based social and health care tools, rather than on effectiveness outcomes or digital tools as end products. Unlike existing reviews, it synthesizes how adaptations are made in practice and which strategies and challenges are reported. These insights can inform developers, researchers, and policymakers seeking to plan more usable, inclusive, and context-sensitive digital transitions in social and health care settings.

User-centered and iterative design approaches were frequently reported across the tools included in this scoping review, describing transitions from paper-based to web-based formats. In the included studies, these approaches were associated with reported improvements in usability, acceptability, or feasibility of the adapted tools. By involving users early and repeatedly throughout the development and usability testing process, developers were able to identify practical challenges and refine digital features accordingly. These approaches are supported by the existing literature. For instance, a recent scoping review by Evans et al [33] reported that digital health interventions developed using user-centered design methodologies were more likely to be perceived as acceptable and feasible in real-world settings, particularly among vulnerable populations. Similarly, a study by Williams and Villa [34] found that involving patients and caregivers in the cocreation process was associated with the development of digital health tools that better aligned with user needs and usability requirements.

This review also showed that even when tools transitioned from paper-based to digital formats, most retained their original content with only modest functional upgrades. Enhancements, such as incorporating multimedia elements or interactive modules, were often aimed to translate the original tool into a digital format rather than redesigning the intervention logic or user pathway. Several tools therefore focused on digitalizing delivery while keeping the core content and structure largely unchanged. At the same time, digital platforms can enable additional functionalities, such as tailored feedback, adaptive navigation, and the integration of user-entered data. Ha et al [35], for example, found that integrating patient-generated health data into digital tools was associated with improved communication and self-management in older adults. In addition, adapting paper-based interventions into digital formats can support accessibility by enabling remote access, self-paced use, and the inclusion of supportive features such as videos or structured guidance. The possibility of tailoring digital health tools to meet users’ needs, such as simplifying language and interface design, can improve engagement and may support better health-related outcomes, particularly among underserved populations [36].

This review also showed that adaptation choices were influenced by the target group. Tools for patients and family caregivers included changes related to usability and accessibility, such as simpler navigation, adapted login systems, and supportive content. Tools for health care professionals more often focused on efficiency, data management, and integration into existing digital workflows. However, these differences were rarely described explicitly, and few studies clearly linked the characteristics of the target group to specific adaptation decisions. Similar patterns are reported in the broader digital health literature, where patient-facing tools focus more on user-centered design and professional tools focus more on workflow integration and efficiency [36]. The limited reporting on target group–specific design decisions suggests that this area needs more systematic attention in future research.

The findings of this review also demonstrate a tension between the intention to improve accessibility and the emergence of new barriers following the transition from paper-based to digital formats. Several tools in our review aimed to extend accessibility by adapting existing tools to an online format. However, this shift also created unintended challenges. For example, usability issues were especially evident in tools targeting older adults or people with limited digital skills, highlighting that increased online availability does not automatically ensure equitable access or sustained use. This mirrors the observations of Veinot et al [37], who argue that digital health innovations often privilege already-resourced users, while vulnerable groups may experience these tools as exclusionary. A systematic review by Crawford and Serhal [38] emphasized that digital transitions must be guided by a digital health equity framework, one that accounts not only for infrastructure and literacy gaps but also for users’ varying emotional readiness and cognitive capacities. Considering these ethical concerns, future development should not focus solely on expanding online availability but should also incorporate inclusive design strategies and, where appropriate, complementary offline options to reduce the risk of widening existing inequities.

Lastly, an important consideration emerging from this review is the need to design digital tools that are flexible enough to function across varied digital environments and resource settings. Some tools included in our scoping review encountered implementation delays or technical issues due to rigid design or integration challenges. To support ongoing usability, digital tools should be built with adaptability in mind (eg, scalable to different systems, compatible with both high- and low-resource contexts, and structured to allow for regular updates as user needs, technologies, and care practices evolve) [39]. As Greenhalgh et al [40] highlight, sustainable digital health interventions must be both technically adaptable and socially responsive to remain relevant and usable over time.

### Strengths and Limitations

This scoping review examined how social health care tools have transitioned from paper-based to web-based formats, a topic that remains relatively underexplored. A key strength of the review is its focus on describing how these transitions were carried out in practice. By analyzing the characteristics of the included tools, such as the design strategies, development approaches, and reported challenges, this study provides insights that may be useful for developers and implementers undertaking similar processes. The inclusion of both academic and gray literature (used to supplement missing information on tools already included) allowed for a more detailed description of the transition processes documented.

However, several limitations should be acknowledged. Many social health care tools may have undergone digital adaptation without formal publication, particularly those developed within routine clinical or community practice. As gray literature was not used to identify additional tools and was used only to supplement information on the included tools, relevant initiatives may not have been captured. In addition, the review was restricted to English-language sources, which may have resulted in the exclusion of tools developed in other linguistic or regional contexts. These limitations point to the need for more transparent and detailed reporting on the transition and implementation of digital health tools.

### Conclusion

This scoping review highlights the importance of considering adaptability when transitioning social health care tools to digital formats, particularly in relation to different systems, resource settings, and user characteristics. Adapting tools to users’ needs, for example, by simplifying navigation, adjusting content presentation, or incorporating interactive elements, may support usability and engagement. The findings suggest that user-centered, iterative, and participatory approaches are commonly used during such transitions and may assist in identifying practical challenges during development and implementation. Continued attention to reporting design decisions and contextual factors will strengthen future research on digital transitions in social and health care settings.

## Supplementary material

10.2196/88631 Multimedia Appendix 1 Search string in peer-reviewed databases.

10.2196/88631 Checklist 1 PRISMA scoping review checklist.

## Abbreviations

PRISMA: Preferred Reporting Items for Systematic Reviews and Meta-Analyses
PRISMA-S: Preferred Reporting Items for Systematic Reviews and Meta-Analyses Literature Search Extension
PRISMA-ScR: Preferred Reporting Items for Systematic Reviews and Meta-Analyses Extension for Scoping Reviews
WSWTTC: What Should We Tell the Children?
