# Supplementary material for: Adaptation of Social Health Care Tools From Paper-Based to Web-Based Formats: Scoping Review
Source: Online J Public Health Inform. 2026 Aug 6;18:e88631. doi: 10.2196/88631 (PMC13446790; doi:10.2196/88631)
Supplement: Supplementary file 2 [file ojphi-v18-e88631-s001.docx]

**Appendix 1 – peer-reviewed databases**

| **Database** | Search Strategy | Hits |
| --- | --- | --- |
| PubMed | ("intervention*" [tiab] OR "Internet-Based Intervention" [Mesh] OR "Psychosocial Intervention" [Mesh] OR "tool*"[tiab] OR "game*" [tiab] OR "Gamification" [Mesh] OR "Gamification" [tiab] OR "program*" [tiab]) AND ("paper-based" [tiab] OR "print-delivered" [tiab] OR "original" [tiab] OR "traditional" [tiab] OR "print-based" [tiab] OR "text-based" [tiab] OR "non-digital" [tiab] OR “printed format*” [tiab] OR “hardcopy” [tiab]) AND ("online" [tiab] OR "digital" [tiab] OR "Digital Health" [Mesh] OR "internet" [tiab] OR "Internet" [Mesh] OR "User-Computer Interface" [Mesh] OR "virtual" [tiab] OR "Virtual Reality" [Mesh] OR web-based [tiab] "Mobile Applications" [Mesh] OR "Application*" [tiab] OR "Computers" [Mesh] OR "Computer*" [tiab]) AND ("communication" [tiab] OR "communication" [Mesh] OR "conversation*" [tiab] OR "discussion*" [tiab] OR "reflective" [tiab] OR "reflection" [tiab] OR "decision-making" [tiab] OR "Decision Making" [Mesh] OR "Decision Making, Computer-Assisted" [Mesh] OR "decision making" [tiab]) AND ("adaptation*" [tiab] OR "Alteration*" [tiab] OR "Transformation*" [tiab] OR "conversion*" [tiab] OR "transition*" [tiab] OR "migration*" [tiab] OR "adjustment*" [tiab] OR "alteration*" [tiab] OR "modification" [tiab] OR "changing" [tiab] OR "replication" [tiab]) | 491 |
| Web of Science | TS=(("intervention*" OR “Internet-Based Intervention” OR “Psychosocial Intervention” OR "tool*" OR "game*" OR “Gamification” OR "program*" OR “Social Support”) AND ("paper-based" OR “Print-Delivered” OR "original" OR "traditional" OR "print-based" OR "text-based" OR "non-digital" OR “printed format*” OR “hardcopy”)) AND ("online" OR "digital" OR "internet" OR "virtual" OR web-based OR “Application*”OR “Computer*”) AND ("communication" OR "conversation*" OR "discussion*" OR "reflective" OR "reflection" OR "decision-making" OR "decision making") AND ("adaptation*” OR “Alteration*” OR “Transformation*” OR “conversion*” OR “transition*" OR “migration*" OR “adjustment*" OR “alteration*" OR “modification" OR “changing" OR “replication") | 486 |
| PsycINFO | (TI,AB,SU("intervention*") OR TI,AB,SU(“Internet-Based Intervention”) OR TI,AB,SU(“Psychosocial Intervention”) OR TI,AB,SU("tool*") OR TI,AB,SU("game*") OR TI,AB,SU(“Gamification”) OR TI,AB,SU("program*") OR TI,AB,SU(“Social Support”)) AND (TI,AB,SU("paper-based") OR TI,AB,SU(“Print-Delivered”) OR TI,AB,SU("original") OR TI,AB,SU("traditional") OR TI,AB,SU("print-based") OR TI,AB,SU("Printed format") OR TI,AB,SU("text-based") OR TI,AB,SU("non-digital") OR TI,AB,SU (“hard-copy”) TI,AB,SU (“printed format*)) AND (TI,AB,SU("online*") OR TI,AB,SU(“digital”) OR TI,AB,SU(“internet”) OR TI,AB,SU(“virtual*") OR TI,AB,SU("web-based") OR TI,AB,SU(“application*”) OR TI,AB,SU("computer*")) AND (TI,AB,SU("communication") OR TI,AB,SU(“conversations”) OR TI,AB,SU("reflective") OR TI,AB,SU("reflection") OR TI,AB,SU("decision-making") OR TI,AB,SU("decision making")) AND (TI,AB,SU("adaptation ") OR TI,AB,SU(“alteration*”) OR TI,AB,SU("transformation*") OR TI,AB,SU("conversion*”) OR TI,AB,SU("transition*") OR TI,AB,SU("migration*") OR TI,AB,SU(“adjustment*") OR TI,AB,SU (“alteration*") OR TI,AB,SU (“modification") OR TI,AB,SU(“changing") OR TI,AB,SU(“replication")) | 198 |
| Scopus | TITLE-ABS-KEY({intervention*} OR {Internet-Based Intervention} OR {Psychosocial Intervention} OR tool* OR game* or Gamification OR program* OR {Social Support}) AND ({paper-based} OR {Print-Delivered} OR {original} OR {traditional} OR {print-based} OR {text-based} OR {non-digital} OR {hard-copy} OR {printed format*}) AND ({online} OR {digital} OR {internet} OR {virtual} OR {web-based} OR {Application*} OR {Computer*”}) AND ({communication} OR {conversation*} OR {discussion*} OR {reflective} OR {reflection} OR {decision-making} OR {decision making}) AND ({adaptation}” OR {alteration*} OR {transformation*} OR {conversion*} OR {transition*} OR {migration*} OR {adjustment*} OR {alteration*} OR {modification} OR {changing} OR {replication}) | 127 |
